# Synchronous Gastric Adenocarcinoma and Diffuse Large B-Cell Lymphoma in the Pelvis: A Rare Case Presentation

**DOI:** 10.1155/2022/7535036

**Published:** 2022-04-28

**Authors:** Evangelia Pliakou, Dimitra-Ioanna Lampropoulou, Nikolaos Soupos, Gerasimos Aravantinos

**Affiliations:** Second Department of Medical Oncology, General Oncology Hospital of Kifissia “Agioi Anargiroi”, Athens, Nea Kifissia 14564, Greece

## Abstract

Multiple primary cancer (MPC) is defined as more than one primary tumour diagnosed at the same patient, either simultaneously or sequentially. Its incidence is low and varies in reporting among medical centers. Diffuse large B-cell lymphoma (DLBCL) is the most common subtype of non-Hodgkin lymphoma (NHL) while gastric cancer (GC) is the fifth most frequently diagnosed malignancy. The aim of this article is to present a rare case of a female patient who was diagnosed with two synchronous malignancies, an adenocarcinoma of the stomach (SRCC) and an aggressive extranodal NH lymphoma (DLBCL) within 2 months. Given the fact that there is an expanding availability of more sensitive diagnostic and screening methods, we aim to increase surveillance amongst medical doctors and provide valuable information for further systematic analysis and identification of such rare cases of concurrent malignancies.

## 1. Introduction

Multiple primary cancer (MPC) is a distinct clinical entity that refers to cases in which more than one primary tumours are diagnosed at the same patient, either simultaneously or sequentially. Synchronous multiple primary cancer (SMPC) is characterised by two or more primary tumours diagnosed within six months [1]. Its incidence is low, and it has been connected with a substantial variation among medical centers; the synchronous presentation of a solid tumour with a hematological malignancy is even less common. Currently, the expanding availability of more sensitive screening methods has contributed to increasing diagnosis of multiple primaries.

Diffuse large B-cell lymphoma (DLBCL), an aggressive type of lymphoma, is the most common subtype of non-Hodgkin lymphoma (NHL), accounting for approximately 30% of all NHL cases [2, 3]. Immunotherapy and chemotherapy remain the most widely used and effective treatment options. Gastric cancer (GC) is the fifth most frequently diagnosed type of malignancy and the fourth cause of cancer death worldwide. In 2020, it newly occurred in 1.09 million people and caused about 769 000 deaths worldwide [4]. The incidence of signet ring cell carcinoma (SRCC) subtype has been constantly increasing, displaying less sensitivity to chemotherapy and a very low 5-year survival rate. Surgical resection and lymphadenectomy remain the gold standard in GC treatment [5]. In this article, we describe a rare case of DLBCL and gastric adenocarcinoma coexisting in an immunocompetent patient; our aim is to increase surveillance amongst medical doctors and provide further information for the diagnosis of SMPC.

## 2. Case Presentation

A 68-year-old female patient presented to a community hospital with a history of epigastralgia, abdominal fullness, and gastroesophageal reflux over the past months. Her medical history was significant for hypothyroidism (under thyroid hormone replacement therapy) and dyslipidemia. The patient was a 45 pack-year smoker (approximately one and a half pack per day). When asked about her family history, she reported that her father was diagnosed with bone cancer while her brother was diagnosed with laryngeal cancer (no further information available). The patient had also suffered from intermittent epigastric discomfort for several years without seeking any medical attention. Fever, night sweats, and weight loss were absent. Upon admission for further medical examination and lab tests, the patient was diagnosed with grade 1 anaemia; none other was significant from the routine hematology and biochemistry tests apart from hepatitis B virus (HBV) surface antigen which resulted positive. Subsequently, the patient was referred for endoscopy: the initial esophagogastroduodenoscopy revealed mucosal edema in the pyloric antrum with the presence of erosion. Furthermore, an irregularly elevated area with edema and redness was noted in the lesser curvature; the *Campylobacter*-like organism test (CLO test) was negative. A second endoscopy, performed about a week later, showed deterioration with some extraphytic ulcerous lesions and greater edema in the lesser curvature. The biopsies revealed a poorly differentiated signet ring cell carcinoma (adenocarcinoma of diffuse type), and the patient was referred to our medical oncology department, for cancer staging and initiation of neoadjuvant therapy. In the meanwhile, the patient suffered from lower back, pelvis, and right hip pain, which was continuously deteriorating to the point that she was unable to walk or stand still (ECOG performance status 3). Magnetic resonance imaging (MRI) and computed tomography (CT) scans were performed, revealing gastric wall thickening in the propyloric area as well as small lymph nodes <10 mm nearby. Furthermore, we found a large, soft-tissue osteolytic mass (97 mm × 76 mm × 97 mm) in the right sacroiliac joint which invaded into the sacrum, the ilium, and the cervical spine at the S1–S4 level. There was no evidence of ascites, peritoneal or liver metastasis. Since adenocarcinoma is not common to metastasize in these areas as an osteolytic lesion, we speculated that the origin of the mass required further investigation. Interestingly, the biopsy from the mass revealed the presence of a lymphoproliferative disease, compatible with DLBCL (CK7−, CD20+, CD-3−, CD138−, CD5−, TDT−, CD10−, bcl6+, MUM1+, bcl2+, c-myk+, ki67: 100%). Thus, the diagnosis of DLBCL with a concurrent gastric adenocarcinoma was confirmed. However, a few days later, while being hospitalized, the patient underwent emergency surgery twice due to gastrointestinal perforation; no treatment was administered due to poor performance status. She was also admitted twice in the intensive care unit (ICU) and eventually died as a result of complications, about three months after the diagnosis of the synchronous malignancies.

## 3. Discussion

According to Fukuda et al., only 16 cases of synchronous gastric cancer and extranodal DLBCL were previously reported; interestingly, only two of them were diagnosed in female patients, like in our case [6]. The underlying mechanisms for developing lymphoma, especially DLBCL, and a concurrent nonhematological malignancy remain unknown. There is, however, evidence supporting a correlation between the development of both tumours; these include immune function alterations, the presence of pathogens, molecular/biological abnormalities, genetic predisposition, or an underlying immunodeficiency. Inaba et al. linked the treatment of primary gastric lymphoma, especially DLBCL, with an increased risk for developing gastric adenocarcinomas [7]. Moreover, an Argentine study concluded that 32% of MPC patients had a family history of cancer [8]. Indeed, our patient had also a significant family history of cancer (both father and brother died of malignancies). Furthermore, other risk factors have been associated with MPCs such as tobacco, alcohol intake, infections (e.g., EBV), and immunosuppression as well as toxic effects related to treatment. The prognosis of MPC patients can be evaluated independently and depends on the stage of each cancer.

Currently, no consensus regarding the management of synchronous gastric adenocarcinoma and lymphoma exists. Thus, decision-making regarding the optimal treatment strategy can be quite challenging. Prioritizing treatment in such patients is crucial, and certain factors must be taken into consideration, such as the stage of each tumour, response to therapy, and performance status. These difficulties in therapy are discussed in detail in a prospective descriptive study conducted by Babu et al., in which lung carcinoma and gastrointestinal malignancies were the most common MPCs [9]. DLBCL typically presents as a rapidly progressing lymphoma, resulting in symptomatic disease that could be fatal without treatment. Thus, DLBCL treatment should be administered immediately upon diagnosis not only due to the aggressive behavior of this malignancy but also because of the high remission rate. Once lymphoma is controlled, treatment for the gastric carcinoma should be initiated based on the patient's condition.

Another topic for consideration is the cumulative toxicities that may develop during MPC treatments; thus, a multidisciplinary approach is crucial for ensuring the optimal effect.

The accurate diagnosis and management of synchronous malignancies can be challenging due to their rare occurrence. Given that it is impractical and not always feasible to obtain a biopsy from every suspicious lesion, the awareness of the different biological behaviors between a gastrointestinal tumour and a lymphoma is critical towards the diagnosis and treatment. After all, the accuracy of the diagnosis is based on the biopsy of the lesion under suspicion. Therefore, if the appearance of new lesions challenges the routine metastatic rule, as happened in our case, the possibility of synchronous multiple primary cancer should be further investigated.

The patient gave fully informed written consent to the publication of this report.

## Data Availability

All the data supporting the conclusions of the study are available upon request.
